# Using a smartphone-based self-management platform to support medication adherence and clinical consultation in Parkinson’s disease

**DOI:** 10.1038/s41531-016-0003-z

**Published:** 2017-11-13

**Authors:** Rashmi Lakshminarayana, Duolao Wang, David Burn, K. Ray Chaudhuri, Clare Galtrey, Natalie Valle Guzman, Bruce Hellman, Ben James, Suvankar Pal, Jon Stamford, Malcolm Steiger, R. W. Stott, James Teo, Roger A. Barker, Emma Wang, Bastiaan R. Bloem, Martijn van der Eijk, Lynn Rochester, Adrian Williams

**Affiliations:** 1 uMotif Ltd, London, UK; 2 Liverpool School of Tropical Medicine, Liverpool, UK; 3 Newcastle-upon-Tyne Hospitals NHS Foundation Trust, Newcastle Upon Tyne, UK; 4 King’s College Hospital NHS Foundation Trust, London, UK; 5 St George’s Healthcare Trust, London, UK; 6 John van Geest Centre for Brain Repair, Cambridge, UK; 7 NHS Forth Valley, Scotland, UK; 8 Cure Parkinson’s Trust, London, UK; 9 The Walton Centre NHS Foundation Trust, Liverpool, UK; 10 King’s College Hospital NHS Foundation Trust, London, UK; 11 John van Geest Centre for Brain Repair & Cambridge University Hospitals NHS Trust, Cambridge, UK; 12 Queen Mary University of London, London, UK; 13 Radboud University Medical Center, Nijmegen, The Netherlands; 14 University Hospitals Birmingham NHS Foundation Trust, Birmingham, UK

## Abstract

The progressive nature of Parkinson’s disease, its complex treatment regimens and the high rates of comorbid conditions make self-management and treatment adherence a challenge. Clinicians have limited face-to-face consultation time with Parkinson’s disease patients, making it difficult to comprehensively address non-adherence. Here we share the results from a multi-centre (seven centres) randomised controlled trial conducted in England and Scotland to assess the impact of using a smartphone-based Parkinson’s tracker app to promote patient self-management, enhance treatment adherence and quality of clinical consultation. Eligible Parkinson’s disease patients were randomised using a 1:1 ratio according to a computer-generated random sequence, stratified by centre and using blocks of variable size, to intervention Parkinson’s Tracker App or control (Treatment as Usual). Primary outcome was the self-reported score of adherence to treatment (Morisky medication adherence scale −8) at 16 weeks. Secondary outcomes were Quality of Life (Parkinson’s disease questionnaire −39), quality of consultation for Parkinson’s disease patients (*Patient-centred questionnaire for Parkinson’s disease*), impact on non-motor symptoms (Non-motor symptoms questionnaire), depression and anxiety (Hospital anxiety and depression scale) and beliefs about medication (Beliefs about Medication Questionnaire) at 16 weeks. Primary and secondary endpoints were analysed using a generalised linear model with treatment as the fixed effect and baseline measurement as the covariate. 158 patients completed the study (Parkinson’s tracker app = 68 and TAU = 90). At 16 weeks Parkinson’s tracker app significantly improved adherence, compared to treatment as usual (mean difference: 0.39, 95%CI 0.04–0.74; *p* = 0.0304) with no confounding effects of gender, number of comorbidities and age. Among secondary outcomes, Parkinson’s tracker app significantly improved patients’ perception of quality of consultation (0.15, 95% CI 0.03 to 0.27; *p* = 0.0110). The change in non-motor symptoms was −0.82 (95% CI −1.75 to 0.10; *p* = 0.0822). 72% of participants in the Parkinson’s tracker app group continued to use and engage with the application throughout the 16-week trial period. The Parkinson’s tracker app can be an effective and novel way of enhancing self-reported medication adherence and quality of clinical consultation by supporting self-management in Parkinson’s disease in patients owning smartphones. Further work is recommended to determine whether the benefits of the intervention are maintained beyond the 16 week study period.

## Digital healthcare: App helps patients stick to plan

A smartphone-based application improves treatment adherence of patients with Parkinson’s disease (PD). Rashmi Lakshminarayana, from uMotif Ltd, and researchers from the UK and The Netherlands describe the results of a 16 week trial of a Parkinson’s tracker app (PTA) that randomised 215 patients from seven different health centres. Patients using the app reported significantly better adherence to their medication plan compared with control patients who continued their treatment as usual. Interestingly, using the PTA also improved the patients’ perception of the quality of their clinical care, possibly as a result of feeling more involved and in control of their care. These findings suggest that by sending medication reminders and tracking symptoms, the app can help patients with Parkinson’s self-manage their increasingly complex treatment regimen as the disease progresses.

## Introduction

Parkinson’s disease (PD) is the second most common neurodegenerative disorder, after Alzheimer’s disease^1^ and affects 6.3 million people worldwide.^2^ It is a disabling condition and has a significant impact on patient’s Quality of Life (QoL).^3^ Non-motor symptoms such as depression, anxiety, fatigue and sleep disturbance are frequently overlooked by clinicians^4^ and contribute to significant burden on people with PD and their carers.^5^ Comorbidity is common in PD. Dementia, arthritis, ischaemic heart disease, diabetes and falls are amongst the commonly associated co-morbidities.^6^

Managing motor and non-motor symptoms and the risk of side effects from medications leads to complex treatment regimes.^7^ The number of drugs and the frequency with which they are taken typically increases as the diseases progresses.^8,9^ The addition of medications to manage associated co-morbidities further adds to the complexity of managing dosing schedules.

Patient centred care—“providing care that is respectful of and responsive to individual patient preferences, needs and values, and ensuring that patient values guide all clinical decisions”^10^—is a core aspect of quality of care and increases treatment adherence among chronically ill patients and job satisfaction among health professionals.^11^ Self-management support and shared decision-making have been identified as two promising ways to support and empower PD patients.^11^

Self-management support refers to increasing patient participation, collaborative goal setting, treatment planning and assisting patients to gain control over their lives.^12^ It can teach PD patients how to better utilise healthcare resources and how to form more effective partnerships with their care providers.^11^

The reported prevalence of non-adherence to prescribed therapy in PD varies from 0 to 60–70%.^1^ Medication nonadherence can be unintentional or intentional.^13^ Unintentional nonadherence involves intending to take a medication as instructed but failing to do so for some reason (e.g. forgetfulness, carelessness) and is influenced by patient characteristics, treatment factors, and patient–provider issues.^14^ Intentional nonadherence involves making a reasoned decision not to take a medication as instructed based on perceptions, feelings or beliefs. It reflects a rational decision-making process by the patient whereby the benefits of treatment are weighed against any adverse effects of the treatment.^14^ The direct and indirect costs of nonadherence in the USA is between $100–$300 billion/year^15^ and over £930 million/year in England.^16^ Nonadherence is linked to poor QoL, increased hospitalisation admissions and premature mortality.^17,18^

Delivering self-management support digitally, including support for understanding and managing treatment has shown improvements health outcomes and processes of care in other chronic conditions.^19^ The SMART-PD trial aimed to assess outcomes of a patient centred smartphone and Internet assisted self-management and treatment adherence tool, the Parkinson’s Tracker App (PTA), to manage PD. Two versions of the PTA were assessed in a previous randomised pilot study in collaboration with the Cure Parkinson’s Trust UK.^20^ Patient feedback on design, features and usability from this study was used to update the PTA for the current trial.

Our primary objective was to assess if patients with PD who use a PTA for 16 weeks in addition to TAU (treatment as usual) show improved self-reported medication adherence. Our secondary objectives were to investigate whether patients who receive the PTA and those who receive TAU differ in terms of QoL, quality of clinical consultation and symptom control.

## Results

**Fig. 1:**
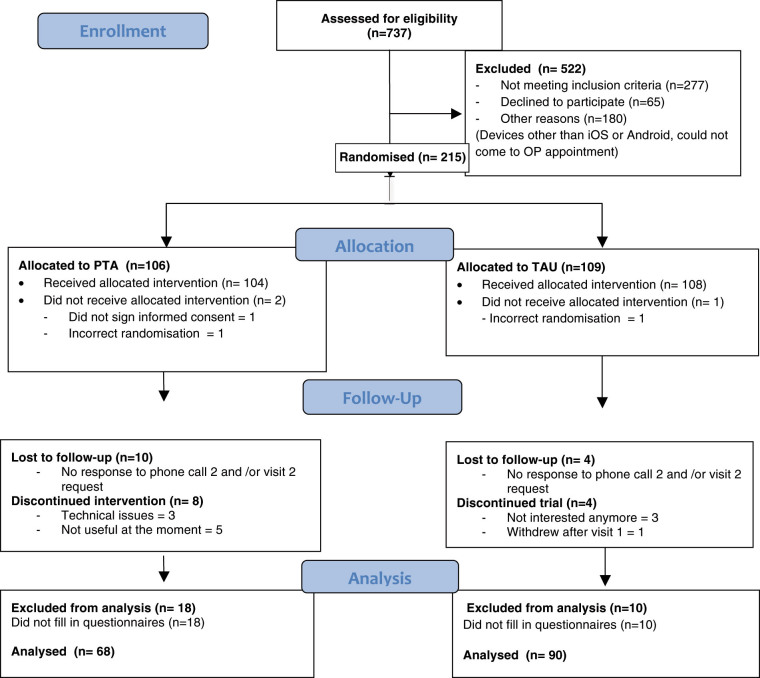
Trial CONSORT patient flow diagram

We invited 737 patients to participate. Of the 522 (70.8%) who responded, 277 (53%) did not meet the inclusion criteria, 65 (12.4%) declined to participate and 180 (34.5%) could not take part due to other reasons including not having devices with iPhone/iPad or Android operating systems or being unable to make it to the Out-Patient appointment. Figure 1 highlights the trial CONSORT flow.

The remaining 215 patients were randomly assigned to PTA (*n* = 106) and control (*n* = 109). Of these, 10 patients (9.4%) and 4 (3.7%) were lost to follow-up in PTA and TAU arm, respectively. Of those who completed the trial, we analysed data from 68 (79%) and 90 (90%) in the PTA and TAU arms, respectively. We closed recruitment due to funding restrictions.

Baseline data were comparable between the two groups (Table 1). Participants took medications for 42 co-morbid conditions in addition to PD (Table 2). Participants took between 1 to 5 types of medications to manage PD. The majority of participants were White (*n* = 206) with a small number being Asian/Asian British (*n* = 6) and Black/Black British (*n* = 2).

**Table 1:** Baseline demographics of participants

Variable	PTA^a^	TAU^b^	All
	(*N* = 94)	(*N* = 107)	(*N* = 201)
	Mean (SD)	Mean (SD)	
Age at screening (year)	59.86 (9.13)	60.71 (10.26)	60.31 (9.73)
Gender			
Female	34 (36.2%)	45 (42.1%)	79 (39.3%)
Male	60 (63.8%)	62 (57.9%)	122 (60.7%)
Number of comorbidities	1.39 (1.66)	1.32 (1.59)	1.35 (1.62)
Parkinson’s disease duration (years)	5.47 (4.18)	5.47 (4.89)	5.47 (4.56)
Morisky Medication Adherence Scale (MMAS-8^c^)	6.03 (1.57)	5.82 (1.48)	5.92 (1.52)
Quality of life (PDQ-39)	154.53 (27.98)	151.43 (27.70)	152.88 (27.81)
Patient-Centered Questionnaire for Parkinson’s Disease (PCQ-PD)	1.91 (0.53)	1.93 (0.51)	1.92 (0.52)
Non-Motor Symptoms Questionnaire (NMSQuest)	10.16 (5.42)	10.24 (5.24)	10.20 (5.31)
Hospital Anxiety Rating Scale (HADSa)	5.49 (3.95)	6.16 (4.14)	5.85 (4.06)
Hospital Depression Rating Scale (HADSd)	5.15 (3.68)	5.14 (3.73)	5.14 (3.70)
Beliefs about Medication Questionnaire (BMQ)	51.83 (9.48)	51.93 (8.09)	51.88 (8.74)
Number who need help with their medication (*n*)	19	27	46

^a^ *PTA* Parkinson’s tracker app

^b^ Treatment as usual

^c^ Use of the ©MMAS is protected by US copyright laws. Permission for use is required. A license agreement is available from: Donald E. Morisky, MMAS Research (MORISKY) 14725 NE 20th St. Bellevue, WA 98007; dmorisky@gmail.com

**Table 2:** Co-morbidities and symptoms for which medications were taken

*Cardiovascular disease*
Hypertension, cardiac arrhythmias (atrial fibrillation), heart failure (congestive heart failure), coronary artery disease (angina, ischaemic heart disease), peripheral vascular disease
*Rheumatology and bone disease*
Osteoporosis, osteoarthritis, gout, dermatomyositis
*Psychological medicine*
Depression, anxiety, panic attacks, psychosis (PD related), insomnia, sleep disturbances, parasomnia (REM sleep disorder), substance abuse / alcohol dependence
*Endocrine disorders*
Hypothyroidism, hyperthyroidism, calcium deficiency, menopause
*Neurological disorders*
Stroke, spasticity, erectile dysfunction, PD related: pain, cramps, excess secretions
*Gastrointestinal disease*
Irritable bowel syndrome (IBS), Crohn’s disease, gastrointestinal reflux/acidity, constipation (PD related), hiatus hernia
*Metabolic disorders* Hypercholesterolaemia, diabetes
*Respiratory disease*
Asthma, sarcoidosis, inflammatory lung disease, sleep apnoea, allergy
*Malignant disease* Prostrate cancer and related urinary urgency
*Haematological disease* Anaemia, vitamin B12 deficiency
*Skin disease* Urticaria
*Eye disorders* Glaucoma
*Supplements* Anti-oxidants, cod liver oil, vitamins
*Others* Hypotension, infection, nausea

### Primary outcome

**Fig. 2:**
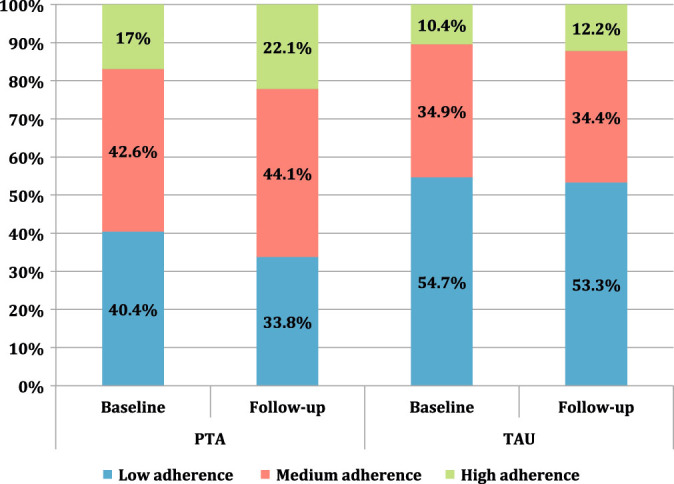
Change in adherence categories: PTA and TAU

Participants in the PTA group (*n* = 68) showed an improvement in MMAS-8^21,22,23^ score of 0.39 points over TAU group in Intent-to-Treat analysis (Table 3) (95% CI 0.04 to 0.74; *p* = 0.0304), translating to better self-reported adherence to medication. These findings were replicated in Per Protocol population (0.41, 95% CI 0.06 to 0.76; *p* = 0.0227). This translated into a 6.6% reduction in low adherence category in the PTA group compared to 1.4% reduction in the TAU group (Fig. 2).

**Table 3:** Change in mean scores on the MMAS-8 between PTA and TAU groups at 16 weeks (intention-to-treat population)

Variable	Statistics/category	PTA^a^	TAU^b^	All
Morisky Medication Adherence Scale (MMAS-8)	*n*	68	90	158
	Mean (SD)	6.30 (1.52)	5.74 (1.53)	5.98 (1.55)
GLM analysis^c^	Difference and 95%CI	0.39 (0.04,0.74)		
	*P*-value	0.0304		
Covariate adjusted GLM analysis^d^	Difference and 95%CI	0.38 (0.03,0.73)		
	*P*-value	0.0331		

^a^ *PTA* Parkinson’s tracker app

^b^ Treatment as usual

^c^ *GLM* generalised linear model

^d^ Age, gender, number of co-morbidities, PD duration were used as covariates. Use of the ©MMAS is protected by US copyright laws. Permission for use is required. A license agreement is available from: Donald E. Morisky, MMAS Research (MORISKY) 14725 NE 20th St. Bellevue, WA 98007; dmorisky@gmail.com

When the ANCOVA analysis was adjusted for the covariates gender, number of comorbidities and age, the effect of treatment remained statistically significant (0.38, 95%CI 0.03 to 0.73; *p* = 0.0301 (Table 3), illustrating that the result of improvement is robust even after controlling for the three covariates on the analysis.

Subgroup analyses with interaction testing were performed to determine whether the improvement in the primary end point was consistent across four important subgroups (Table 4). No significant interactions were observed. However, results revealed that those older than 61, females and those with greater than one comorbidity tended to benefit more from PTA than their counterparts.

**Table 4:** Comparison of age at screening, gender, number of co-morbidities and duration of Parkinson’s disease between PTA and TAU groups

		*n*, mean (SD)		GLM analysis	
Subgroup variable	Category	PTA	TAU	Difference and 95%CI	*p*-value
Age at screening (year)					
	≤61	33,6.08 (1.49)	45,5.69 (1.53)	0.08 (−0.44,0.59)	0.7752
	>61	35,6.51 (1.54)	45,5.79 (1.54)	0.70 (0.23,1.17)	0.0036
Gender					
	Male	43,6.33 (1.52)	52,5.86 (1.49)	0.29 (−0.17,0.75)	0.2108
	Female	25,6.24 (1.56)	38,5.58 (1.58)	0.51 (−0.03,1.04)	0.0636
Number of comorbidities					
	≤1	43,6.13 (1.64)	59,5.94 (1.45)	0.21 (−0.18,0.60)	0.2892
	>1	25,6.59 (1.26)	31,5.37 (1.63)	0.84 (0.15,1.54)	0.0167
Parkinson’s disease duration (year)					
	≤4	37,6.56 (1.30)	52,5.88 (1.55)	0.47 (−0.03,0.96)	0.0630
	>4	31,5.98 (1.71)	38,5.56 (1.50)	0.33 (−0.17,0.83)	0.1917

*GLM* generalised linear model, *PTA* Parkinson’s tracker app, *TAU* treatment as usual

### Secondary outcomes

Among the secondary outcomes (Table 5) the PCQ-PD improved in the PTA group (*p* = 0.0110). Quality of PD care was assessed in 6 dimensions in PCQ-PD (i.e. information, collaboration, accessibility, empathy, patient involvement and emotional support). Statistically significant improvements were seen in patient perception of collaboration and patient involvement in decision-making subscales.

**Table 5:** Difference in mean scores on secondary outcomes between PTA and TAU groups at 16 weeks (intention-to-treat population)

	*n*, mean (SD)		GLM analysis	
Variable	PTA	TAU	Difference and 95%CI	*p*-value
QoL (PDQQoL-39)	68,155.47 (28.16)	89,150.75 (25.62)	−0.22 (−3.95,3.52)	0.9102
Patient-Centred Questionnaire for Parkinson’s Disease (PCQ-PD)	68,2.03 (0.48)	89,1.85 (0.54)	0.15 (0.03,0.27)	0.0110
Non-Motor Symptoms Questionnaire (NMSQuest)	68,9.82 (5.68)	89,10.66 (4.89)	−0.82 (−1.75,0.10)	0.0822
Hospital Anxiety Rating Scale (HADSa)	68,6.03 (4.15)	89,6.31 (4.21)	0.30 (−0.42,1.01)	0.4136
Hospital Depression Rating Scale (HADSd)	68,5.26 (3.73)	89,5.53 (3.90)	0.03 (−0.63,0.70)	0.9195
Beliefs about Medication Questionnaire (BMQ)	68,51.84 (8.85)	89,52.19 (7.97)	−0.42 (−2.16,1.32)	0.6355

*GLM* generalised linear model, *PTA* Parkinson’s tracker app, *TAU* treatment as usual

The difference between the PTA and TAU groups for the secondary endpoint of Non-Motor Symptom Questionnaire was −0.82 (*p* = 0.0822) and for the QoL questionnaire (PDQ-39) was −0.22 (*p* = 0.9102). Results for individual subdomains of PDQ-39 are presented in Supplementary Table 1.

There were no statistically significant differences between the groups with regards to other secondary outcomes. These findings were consistent with per protocol analysis.

### Analysis of PTA use

Seventy-two per cent of participants in the PTA group continued to use and engage with the application throughout the 112-day trial period (Table 6). They used the app almost every other day on average, with some people using the app every day (Table 7).

**Table 6:** PTA user retention over trial period (August 2014–June 2015) (*n* = 68)

	Proportion of trial complete			
	25%	50%	75%	100%
Number of days used	28	56	84	112
Number of patients using app	56	56	53	49
Proportion of patients retained	82%	82%	78%	72%

**Table 7:** PTA use frequency and volume of data input

App usage frequency during study period	No. of days
Average number of days tracked during study period	48
Maximum number of days tracked	113
App data input volume during study period	Number
Average no. of symptom scores entered in motif interface	629
Maximum no. of symptom scores entered	3608
Median no. of symptom scores entered	565

We undertook further analysis of PTA usage in the PTA group in March 2016, after the end of the study in June 2015. The table below (Table 8) highlights that 29% of participants continued to use the app for over 6 months after the trial had ended.

**Table 8:** PTA user retention after trial period (as of March 2016)

	No. of participants
Tracking for >182 days (i.e. >6 months)	18
Tracking for >365 days (i.e. >12 months)	3
	Days
Longest use recorded	510

## Discussion

Compared to TAU, participants using the PTA for 16 weeks reported statistically significant improvements in short-term self-reported medication adherence and subjective quality of clinical consultation. These findings suggest that the PTA can be useful in improving outcomes and processes of care in people with PD, similar to results seen in other chronic conditions such as diabetes and asthma.^19^ Given that typical clinical follow-up of patients with PD by clinical care teams in the UK occurs approximately every 6 months (by consultants or nurse specialists), this finding suggests PTA is useful in improving care for a significant period of time between clinic consultations.^7^

In early stages of the disease, patients usually take a single drug^24^ and over half of patients take two to three drugs three to four times daily in later stages.^9,25^ This partly explains why the effect of the PTA in subgroups was significant amongst those older than the median age. The complexity of treatment along with PD affecting cognitive processes such as sorting or planning tasks^26^ might explain why the PTA helped in improving adherence as medication reminders and symptom tracking were core features.

Factors affecting medication non-adherence in PD patients include clinical (i.e. mood disorders, cognitive impairment, poor symptom control or reduced QoL, younger age or longer disease duration, and regime complexity or polypharmacy) and demographic (lack of spouse or partner, low income, employment status, and gender) factors.^27^ We only excluded those with significant cognitive impairment as diagnosed by the clinical team. Baseline HADS scores suggested that a majority of the participants did not have depression or anxiety. There was an indication that women were more adherent than men, but this was not statistically significant. The average age and duration of PD was 61 years and 4 years respectively. Most participants took all their medications on their own with 20.2% (*n* = 19) in the PTA group and 25.2% (*n* = 27) in the TAU group needing help to take their medications.

Composite QoL scores as measured on the PDQ-39 were low at baseline, indicating that this was not a significant contributor to the outcome. Further investigation is recommended on the impact of improved adherence on non-motor symptoms.^28^ However, this could be a chance finding or a placebo effect, as we had not included a formal non-motor symptom assessment.

PD patients desire more emotional support from healthcare professionals and want more active involvement in clinical decision-making.^11^ Other studies have found that PD patients who perceived higher involvement in their care were more satisfied with the consultation and intended to be more compliant with treatment.^29^ Those in the PTA group perceived the service as more patient-centred as measured on the PCQ-PD (*p* = 0.01). This could be linked to clinical teams inviting patients to use the app, personalising the app set-up and jointly reviewing progress using the data collected by patients at the 16-week follow-up.

User retention is a recognised challenge for all Smartphone apps, with less than one in seven people opening apps beyond a day after download.^30^ The higher level of user retention during the SMART-PD trial can be linked to factors including the simplicity and design of the app’s user interface and user experience; that the app is perceived as given by participant’s clinical team and thereby acting as an extension of their recommended care; and the direct benefit of understanding their health better as reported by PTA users.

The trial included only those who already had smartphones or tablet devices, thereby excluding those who did not own such a device. Smartphone penetration in the UK is estimated at 66%, ranging from 88% in those aged 25–34 to 49% in those aged 55–64 and 17% in those over 65.^31^ These later groups are the most represented in the study group. The study app was available on iOS (Apple operating system) and Android (Google operating system). These were the major operating systems during the study period (August 2014–June 2015) with 42.33% being iOS users and 47.95% being Android users. We estimate that users of other operating systems (i.e. 9.72% of total smartphone and tablet device users during the study period used Windows and Blackberry)^32^ could not take part, further contributing to the reported response rate. As the smartphone market penetration increases, especially in those over 65, it would be anticipated that more patients would be able to use the study app. It should be noted that a mean age of 60 in the PTA group was not a barrier for interaction with the PTA, allaying fears that technology-based interventions are not appropriate for an older age group.

Our results also clearly highlight the difficulty of studying adherence using a pragmatic design. Research into recruitment rates for randomised controlled trials (RCTs)^33^ highlight the challenges of both recruiting patients and of retentaining recruited patients despite implementing various retention strategies along with letters. Since we did not use any other methods of retention, this could explain the study retention rate we found in the study—there was a higher than expected loss to follow-up and survey completion rates (64% in PTA arm and 83% in the TAU arm completed as Per Protocol). We relied on the use of online questionnaires that patients completed at home, rather than a standard paper-based questionnaire completed at the clinic during study visits, which likely led to the rate of drop-out. However, questionnaires delivered and completed by the patient at home have the benefit of reduced recall bias and in-situ data collection. It is possible that follow-up drop-outs preferentially affect those with poor self-reported medication adherence, the difference in medications adherence between study arms were maintained in both intention-to-treat and per-protocol analysis. The higher study drop-out rate in the PTA group is likely due to the participants needing to perform an additional trial task of using the PTA compared to the TAU group. Additionally, the PTA may not have met all participants’ needs (for example: some of the self-monitoring measures chosen by the trial team may not be applicable to some participants), which could have contributed to the dropouts.

The MMAS-8 was developed to manage hypertension^20^ and the MMAS-4 has been used in PD studies.^34^ A 2-point improvement on the MMAS-8 scores was found to link to clinically significant improvement in hypertension.^35^ However, there are no similar measures of the MMAS-8 in PD. Some studies investigating adherence chose participants with lower adherence^36^ as criteria for inclusion in a trial, which translates to an increased potential for improvement. We did not exclude patients with high self-reported adherence, indicating that PTA can help a range of people with varying levels of adherence.

To study the impact of the PTA on health services use, we need to assess the PTA over a longer duration. Although it seems reasonable to conclude that improvement in adherence is desirable, specifics around how much self-reported adherence is clinically meaningful in PD will require additional studies looking specifically at clinician and carer measures.

In summary, we conclude that the PTA could be an effective and novel way of enhancing short-term self-reported medication adherence and quality of clinical consultation by supporting self-management in PD.

## Methods

An open-label, multicentre RCT was conducted from August 2014 to June 2015. The objective was to compare the use of smartphone and internet-enabled PTA with TAU for 16 weeks among patients with PD across 7 centres in England and Scotland (John van Geest Centre for Brain Repair, Cambridge University Hospitals NHS Trust, Cambridge UK; King’s College Hospital NHS Foundation Trust, London, UK; Newcastle upon Tyne Hospitals NHS Foundation Trust, Newcastle, UK; NHS Forth Valley, Scotland, UK; St. George’s Healthcare NHS Trust, London, UK; The Walton Centre NHS Foundation Trust, Liverpool, UK ; University Hospitals Birmingham NHS Foundation Trust, Birmingham, UK.) Ethics was obtained from the National Research Ethics Service London–Westminster Research Ethics Committee (13/LO/1783). Details of the trial are available in the trial protocol.^37^

Eligibility criteria were drawn up in a pragmatic manner to demonstrate both the effectiveness and the ease of implementation of the PTA if used in routine clinical practice.

Inclusion criteria included, (i) a diagnoses of probable, idiopathic PD, (ii) on one or more Parkinson’s medications not altered within the previous month and not expected to change during the trial period, and, (iii) English-speaking and literate with access to a smartphone and/or tablet or internet on a daily basis at home. Key exclusion criteria included (i) suspected Parkinsonism due to causes other than idiopathic PD, (ii) current or previous treatment for side effects of prolonged neuroleptic treatment and, (iii) a diagnosis of dementia or significant cognitive impairment or major psychiatric illness associated with psychosis or a major, serious comorbid illness (as recorded in patient case file).

### Parkinson’s tracker app

The clinical team identified potentially eligible patients across the seven trial sites from the clinic lists 6 weeks prior to upcoming outpatient (OP) appointments. An information pack containing a patient invitation letter, a participant information sheet and a consent form was sent to potential participants 3–4 weeks prior to their next OP clinic appointment. At the OP appointment, the clinician rechecked eligibility criteria and administered the informed consent.

Following consent, patients were randomised to either the PTA or the TAU group. They completed the baseline questionnaires at the clinic or were asked to complete it at home within 1 week of their OP appointment and were given the date for follow-up. Apart from consent forms, no paper-based questionnaires or forms were used.

Patient questionnaires were administered online using a secure research-grade questionnaire tool (Qualtrics) and other data was collected using site specific OpenClinica electronic Research Form.

Those allocated to the PTA arm received instructions from their clinical team to download the PTA to their Android or iPhone smartphones or tablet devices (using the free ‘uMotif’ app from the Google Play Store or Apple App Store) or to access it via a website portal within 1 day after they have attended their OP appointment.

The app consisted of the following features:

1. A sliding petal interface to track ten self-monitoring measures, chosen by the trial team, on a 5-point scale: including sleep, exercise, mood, energy, movement, suppleness (Fig. 3). An accessibility mode with a zoom function to magnify the screen was included.

**Fig. 3:**
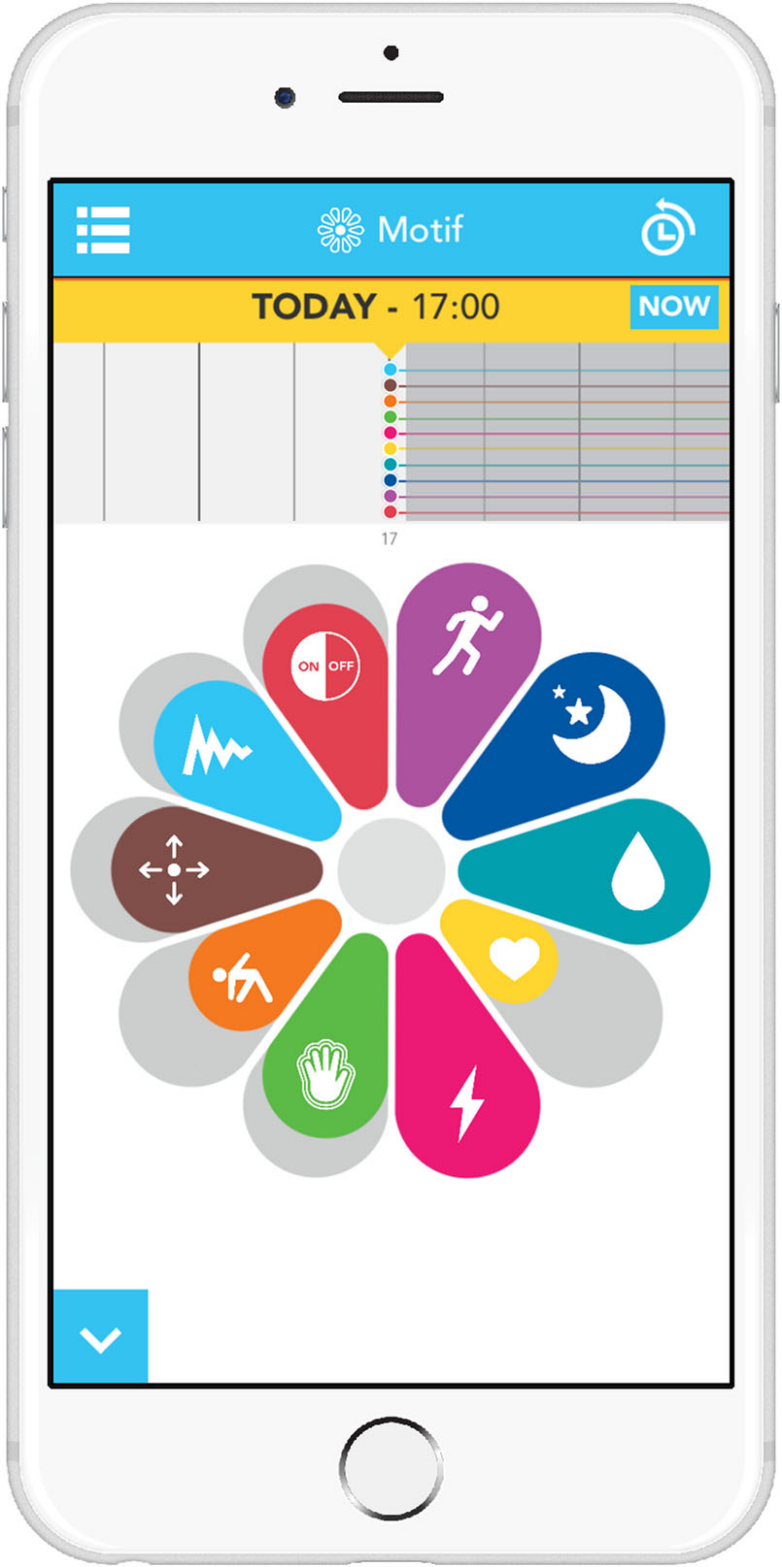
Screenshot of PTA self-tracking interface
2. A reminder system for patients to set up to allow the patient to receive alerts to help track medication (Fig. 4)

**Fig. 4:**
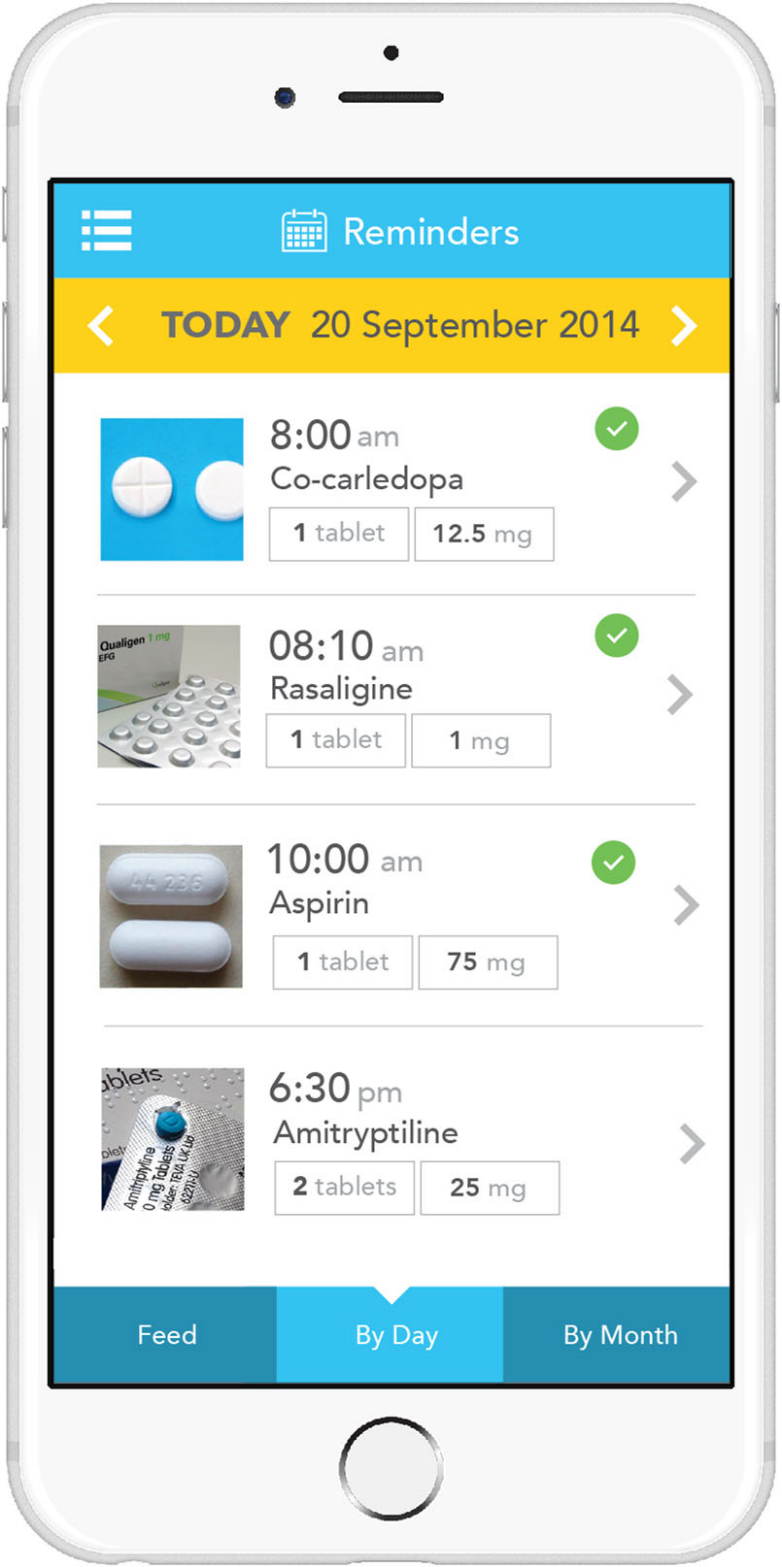
Screenshot of PTA medication reminders
3. An option to generate a report of data entered by the patient over the trial period as an aid at their 16 week follow-up appointment (Fig. 5)

**Fig. 5:**
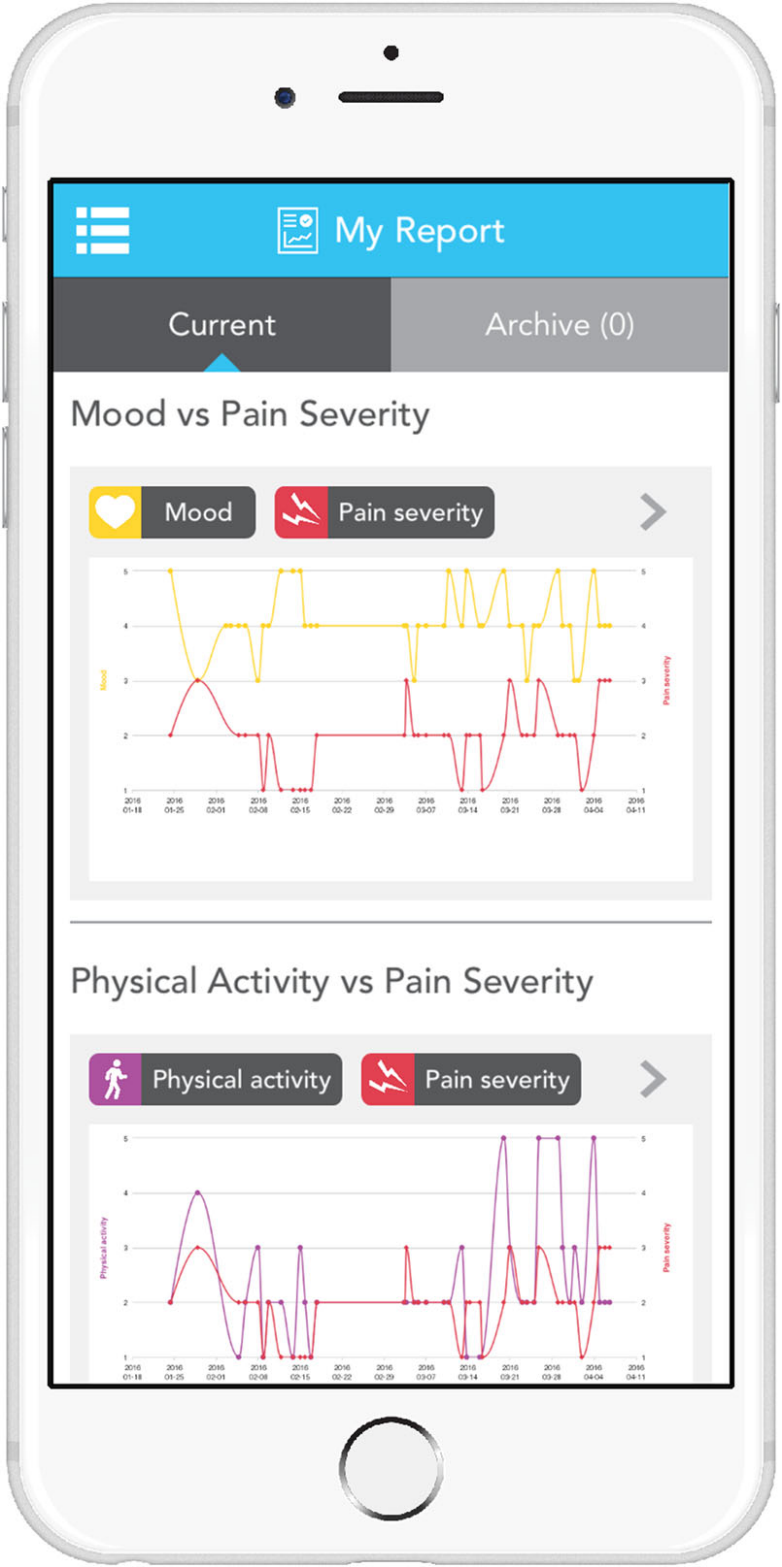
Screenshot of PTA report
4. Games to track physical responsiveness (finger-tapping task) (Fig. 6) and cognition (number-size Stroop test)

**Fig. 6:**
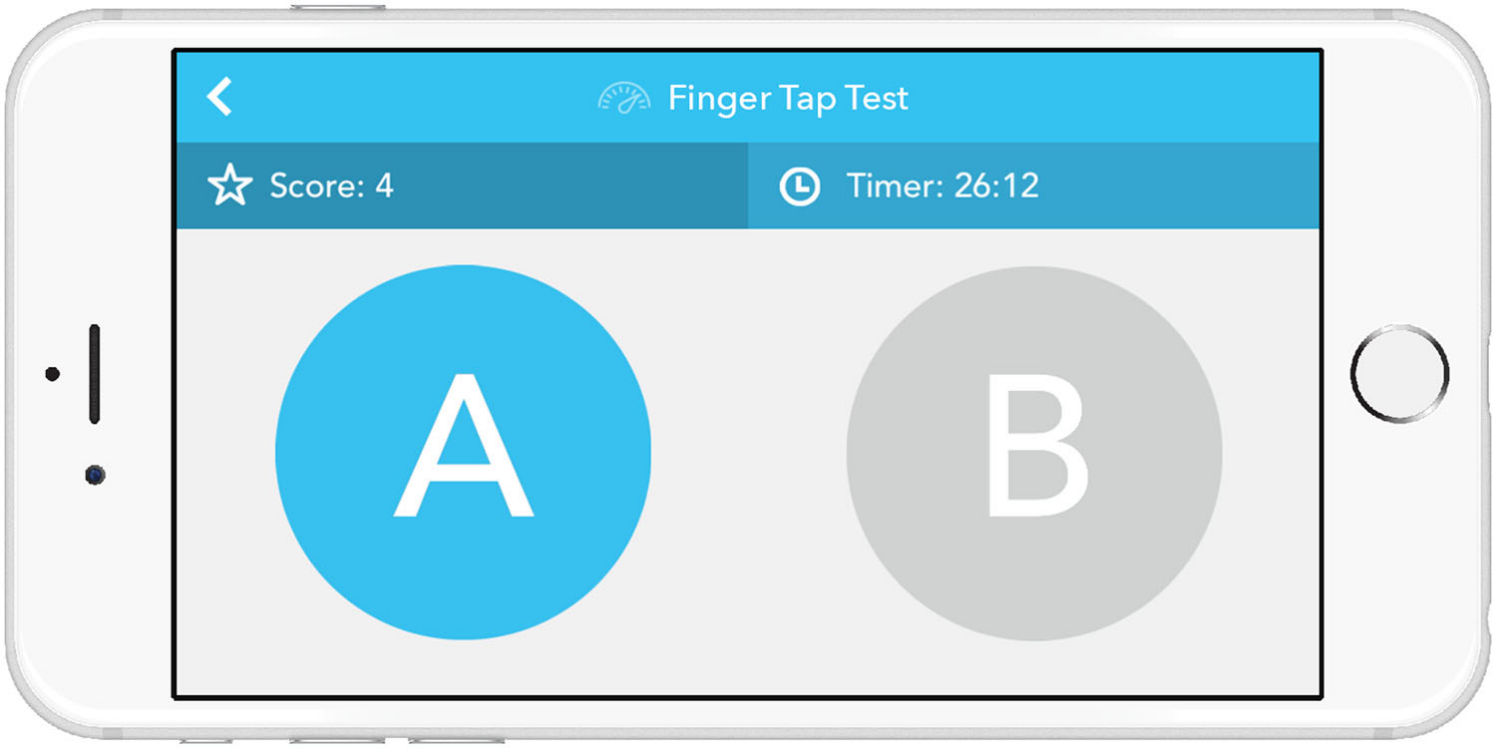
Screenshot of PTA finger tapping test
5. Information about PD from Parkinson’s UK and the Cure Parkinson’s Trust

Participants were asked to set up medication reminders and use the app once a day or if not possible at least on alternate days over 16 weeks. They also received a call from their clinician after 2 weeks to check if they, (i) had completed the baseline study questionnaires, (ii) had downloaded the app and, (iii) were having any difficulties with the PTA. At the 16-week visit participants used the data they collected on their app during their consultation. Technical support was available for patients via email throughout the study if they experienced problems with the PTA.

Participants in the TAU group received a call from their clinicians after 2 weeks to check if they had completed the study questionnaires. At the 16 weeks appointment, they had their regular OP clinical assessments, including symptom review followed by a medication review. Participants in the TAU group were offered access and use of the PTA after their 16 weeks of TAU

Participants in both groups received a reminder phone call from their clinician 1 to 2 weeks prior to the follow-up appointment. Participants completed the end of trial questionnaires within 1 week after their 16-week follow-up appointment.

Participants in both arms had 2 contacts at the clinic. Visit 1 included talking through the consent, randomisation and giving access to the app if allocated to the intervention. This took between 30–45 min Visit 2 at the end of the study period included reviewing the data collected in the app for the intervention and for control included inviting to the app and lasted 30 min

Recruitment was originally planned as a rolling programme over the course of 16 weeks until recruitment targets are achieved. However, we extended the recruitment by another 6 months to help recruit the required patient numbers.

### Outcome measures

The primary outcome measure was self-reported adherence to treatment as determined by Morisky Medication Adherence Scale (MMAS-8).^38^ This is a self-report eight-item scale with response categories of ‘yes’ or ‘no’ for each item and a 5-point Likert response for the last item. Higher responses indicate better adherence. Secondary outcome measures included:

1. Parkinson’s Disease Questionnaire (PDQ-39):^39^ Measures eight dimensions of QoL–mobility, activities of daily living, emotional wellbeing, stigma, social support, cognition, communication and bodily discomfort. Lower total scores reflect better QoL.
2. Patient-Centered Questionnaire for PD:^40^ (PCQ-PD) to measure changes in quality of consultation. PCQ-PD is a self–completed questionnaire addressing forty-four care aspects in six subscales (information, multidisciplinary collaboration, accessibility, empathy, patient involvement and emotional support) of patient-centeredness.
3. Non-Motor Symptom Questionnaire:^41^ A 30-item self-completed questionnaire featuring responses as “yes,” or “no” to each item.
4. Hospital Anxiety and Depression Scale:^42^ A self-screening questionnaire for anxiety and depression. It consists of 14 questions with seven each for anxiety and depression.
5. Beliefs about Medication Questionnaire:^43^ A self-administered questionnaire and comprising of two subscales: An 11-item questionnaire relating to prescribed medication and an 8-item questionnaire relating to general views about taking medication. Respondents rate each item on a five point Likert-type scale depending on their degree of agreement (1 strongly disagree, 5 strongly agree). Higher scores indicate higher levels of concern or strong beliefs towards the use of medication.

All measures were collected at baseline and at end of the trial period of 16 weeks.

### Sample size

The sample size calculation was based on the primary endpoint. To detect a 1-point improvement on the MMAS-8 with a standard deviation (SD) of 2.5 and 80% power at the 5% significance level would require 200 subjects (100 in each group, 1:1 allocation). To allow for 10% loss due to dropouts and those lost to follow-up, we aimed to recruit 222 subjects (111 in each group, 1:1 allocation).

### Randomisation and masking

Eligible patients were allocated in a 1:1 ratio to the two arms of the study according to a computer-generated random sequence stratified by centre and using blocks of variable size. The allocation sequence was generated by an independent statistician and was not available to any member of the research team until databases were completed and locked. Copies of the allocation sequence were not held at the recruiting centres. Once randomised, clinicians who enroled participants informed them of their assignment. Blinding to group allocation was not possible, as participants knew whether or not they were receiving PTA. The trial statisticians were blinded to the treatment code when developing the statistical analysis plan and writing the statistical programmes, which were validated and completed using dummy randomisation codes. The actual allocation code was only provided to the trial statisticians after locking of the database and finalisation of the statistical analysis plan.

### Statistical methods

A Generalised Linear Model (GLM) was used for analysis of the primary endpoint and had treatment as the fixed effect and baseline measurement of the primary endpoint as the covariate. The treatment difference together with its 95% confidence interval between PTA and TAU in the least square mean of primary endpoint was derived from the GLM model. Model assumptions about residuals in regression analysis were checked by inspection of residuals vs. a fitted values plot and no serious violation of normality assumption was found. In addition, adjusted analysis and subgroup analysis with pre-specified covariates (age, gender and number of co-morbidities) were performed. For the secondary outcomes (QoL, depression, anxiety, non-motor symptoms, and degrees of depression and anxiety and quality of consultation), the analyses were performed in an analogous fashion within the framework of GLM. Analyses of the primary and secondary outcomes were carried out in adherence to the intention-to-treat principle. In addition, supplemental per-protocol analyses were performed.

## Electronic supplementary material

Supplementary Information
