# Author Correction: Using a smartphone-based self-management platform to support medication adherence and clinical consultation in Parkinson’s disease

**DOI:** 10.1038/s41531-017-0034-0

**Published:** 2017-11-13

**Authors:** Rashmi Lakshminarayana, Duolao Wang, David Burn, K. Ray Chaudhuri, Clare Galtrey, Natalie Valle Guzman, Bruce Hellman, Ben James, Suvankar Pal, Jon Stamford, Malcolm Steiger, R. W. Stott, James Teo, Roger A. Barker, Emma Wang, Bastiaan R. Bloem, Martijn van der Eijk, Lynn Rochester, Adrian Williams

**Affiliations:** 1 uMotif Ltd, London, UK; 2 Liverpool School of Tropical Medicine, Liverpool, UK; 3 Newcastle-upon-Tyne Hospitals NHS Foundation Trust, Newcastle-upon-Tyne, UK; 4 King’s College Hospital NHS Foundation Trust, London, UK; 5 St George’s Healthcare Trust, London, UK; 6 John van Geest Centre for Brain Repair, Cambridge, UK; 7 NHS Forth Valley, Scotland, UK; 8 Cure Parkinson’s Trust, London, UK; 9 The Walton Centre NHS Foundation Trust, Liverpool, UK; 10 King’s College Hospital NHS Foundation Trust, London, UK; 11 John van Geest Centre for Brain Repair & Cambridge University Hospitals NHS Trust, Cambridge, UK; 12 Queen Mary University of London, London, UK; 13 Radboud University Medical Center, Nijmegen, The Netherlands; 14 University Hospitals Birmingham NHS Foundation Trust, Birmingham, UK

**Correction to:** *npj Parkinson's Disease* (2017); doi:10.1038/s41531-016-0003-z; Published online 09 January 2017

**Table 1:** Baseline demographics of participants

Variable	PTA^a^	TAU^b^	All
	(*N* = 94)	(*N* = 107)	(*N* = 201)
	Mean (SD)	Mean (SD)	
Age at screening (year)	59.86 (9.13)	60.71 (10.26)	60.31 (9.73)
Gender			
Female	34 (36.2%)	45 (42.1%)	79 (39.3%)
Male	60 (63.8%)	62 (57.9%)	122 (60.7%)
Number of comorbidities	1.39 (1.66)	1.32 (1.59)	1.35 (1.62)
Parkinson’s disease duration (years)	5.47 (4.18)	5.47 (4.89)	5.47 (4.56)
Morisky Medication Adherence Scale (MMAS-8^c^)	6.03 (1.57)	5.82 (1.48)	5.92 (1.52)
Quality of life (PDQ-39)	154.53 (27.98)	151.43 (27.70)	152.88 (27.81)
Patient-Centered Questionnaire for Parkinson’s Disease (PCQ-PD)	1.91 (0.53)	1.93 (0.51)	1.92 (0.52)
Non-Motor Symptoms Questionnaire (NMSQuest)	10.16 (5.42)	10.24 (5.24)	10.20 (5.31)
Hospital Anxiety Rating Scale (HADSa)	5.49 (3.95)	6.16 (4.14)	5.85 (4.06)
Hospital Depression Rating Scale (HADSd)	5.15 (3.68)	5.14 (3.73)	5.14 (3.70)
Beliefs about Medication Questionnaire (BMQ)	51.83 (9.48)	51.93 (8.09)	51.88 (8.74)
Number who need help with their medication (*n*)	19	27	46

^a^ *PTA* Parkinson’s tracker app

^b^ Treatment as usual

^c^ Use of the ©MMAS is protected by US copyright laws. Permission for use is required. A license agreement is available from: Donald E. Morisky, MMAS Research (MORISKY) 14725 NE 20th St. Bellevue, WA 98007; dmorisky@gmail.com

**Table 3:** Change in mean scores on the MMAS-8 between PTA and TAU groups at 16 weeks (intention-to-treat population)

Variable	Statistics/category	PTA^a^	TAU^b^	All
Morisky Medication Adherence Scale (MMAS-8)	*n*	68	90	158
	Mean (SD)	6.30 (1.52)	5.74 (1.53)	5.98 (1.55)
GLM analysis^c^	Difference and 95%CI	0.39 (0.04,0.74)		
	*P*-value	0.0304		
Covariate adjusted GLM analysis^d^	Difference and 95%CI	0.38 (0.03,0.73)		
	*P*-value	0.0331		

^a^ *PTA* Parkinson’s tracker app

^b^ Treatment as usual

^c^ *GLM* generalised linear model

^d^ Age, gender, number of co-morbidities, PD duration were used as covariates. Use of the ©MMAS is protected by US copyright laws. Permission for use is required. A license agreement is available from: Donald E. Morisky, MMAS Research (MORISKY) 14725 NE 20th St. Bellevue, WA 98007; dmorisky@gmail.com

In the original version of this article the copyright notice was missing from Tables 1 and 3. This has now been added alongside the three relevant references inserted as refs. 21–23. The correction has been published and is appended to both the HTML and PDF versions of this paper. The errors have been fixed in the paper.

New references inserted:

Participants in the PTA group (n = 68) showed an improvement in MMAS-8.^21,22,23^

21. Morisky, D. E., Ang, A., Krousel-Wood, M., Ward, H. Predictive validity of a medication adherence measure for hypertension control. *J. Clin. Hypertens*. **10**, 348–354 (2008).

22. Krousel-Wood, M. A. et al. New medication adherence scale versus pharmacy fill rates in seniors with hypertension. *Am. J. Manag. Care* **15**, 59–66 (2009).

23. Morisky, D. E., DiMatteo, M. R. Improving the measurement of self-reported medication nonadherence: final response. *J Clin Epidemio* **64**, 258–263 (2011). PMID:21144706.

