# Supplementary material for: Using a smartphone-based self-management platform to support medication adherence and clinical consultation in Parkinson’s disease
Source: NPJ Parkinsons Dis. 2017 Nov 13;3:2. doi: 10.1038/s41531-016-0003-z (PMC5460235; doi:10.1038/s41531-016-0003-z)
Supplement: Supplementary file 1 — Supplementary Information [file 41531_2016_3_MOESM1_ESM.docx]

**Supplementary Table 1: Analysis of PDQ-39 subscales between PTA and TAU groups at 16 weeks (intention-to-treat population)**

|  | **mean (SD)** | | **GLM*** analysis** | |
| --- | --- | --- | --- | --- |
| **Variable** | **PTA*** | **TAU**** | **Difference and 95%CI** | **p-value** |
| PDQ-39 Quality of Life: Mobility | 25.51 (23.41) | 30.83 (26.89) | 0.36(-3.58,4.30) | 0.8576 |
| PDQ-39 Quality of Life: Activities of Daily Living | 24.69 (20.25) | 28.01 (21.03) | -0.61(-3.97,2.75) | 0.7235 |
| PDQ-39 Quality of Life: Emotional wellbeing | 24.14 (20.14) | 25.88 (20.64) | 1.26(-2.57,5.08) | 0.5204 |
| PDQ-39 Quality of Life: Stigma | 23.16 (24.15) | 22.85 (20.01) | 2.45(-1.37,6.28) | 0.2089 |
| PDQ-39 Quality of Life: Social support | 14.71 (19.69) | 12.55 (16.48) | 1.64(-2.15,5.44) | 0.3964 |
| PDQ-39 Quality of Life: Cognition | 25.55 (19.73) | 31.39 (19.40) | -4.21(-7.82,-0.59) | 0.0225 |
| PDQ-39 Quality of Life: Communication | 22.55 (23.75) | 27.15 (22.53) | -0.91(-4.53,2.71) | 0.6214 |
| PDQ-39 Quality of Life: Bodily discomfort | 44.49 (28.09) | 47.38 (23.69) | -1.87(-6.62,2.89) | 0.4421 |
| PDQ-39 Quality of Life: Single index of all scores | 25.60 (17.62) | 28.20 (15.06) | -0.04(-2.30,2.23) | 0.9754 |

*PTA=Parkinson’s Tracker App

**Treatment as Usual

***GLM = Generalised Linear Model
